# Structural analysis of the human SYCE2–TEX12 complex provides molecular insights into synaptonemal complex assembly

**DOI:** 10.1098/rsob.120099

**Published:** 2012-07

**Authors:** Owen R. Davies, Joseph D. Maman, Luca Pellegrini

**Affiliations:** Department of Biochemistry, University of Cambridge, 80 Tennis Court Road, Old Addenbrookes Site, Cambridge CB2 1GA, UK

**Keywords:** synaptonemal complex, meiosis, homologous recombination, central element, SYCE2, TEX12

## Abstract

The successful completion of meiosis is essential for all sexually reproducing organisms. The synaptonemal complex (SC) is a large proteinaceous structure that holds together homologous chromosomes during meiosis, providing the structural framework for meiotic recombination and crossover formation. Errors in SC formation are associated with infertility, recurrent miscarriage and aneuploidy. The current lack of molecular information about the dynamic process of SC assembly severely restricts our understanding of its function in meiosis. Here, we provide the first biochemical and structural analysis of an SC protein component and propose a structural basis for its function in SC assembly. We show that human SC proteins SYCE2 and TEX12 form a highly stable, constitutive complex, and define the regions responsible for their homotypic and heterotypic interactions. Biophysical analysis reveals that the SYCE2–TEX12 complex is an equimolar hetero-octamer, formed from the association of an SYCE2 tetramer and two TEX12 dimers. Electron microscopy shows that biochemically reconstituted SYCE2–TEX12 complexes assemble spontaneously into filamentous structures that resemble the known physical features of the SC central element (CE). Our findings can be combined with existing biological data in a model of chromosome synapsis driven by growth of SYCE2–TEX12 higher-order structures within the CE of the SC.

## 2. Introduction

Human fertility and genetic diversity depend on the successful execution of the genetic programme of meiosis. At the physical and functional centre of meiosis is the synaptonemal complex (SC), an enigmatic proteinaceous superstructure that holds together homologous chromosome pairs, providing the structural framework within which meiotic recombination and crossover formation occur [1–5]. The SC is essential for the successful completion of meiotic cell division: its disruption in mice leads to complete meiotic failure and resultant infertility [6–10], and its defective function in humans is associated with infertility and recurrent pregnancy loss (affecting 15% and 5% of couples, respectively), in addition to non-lethal aneuploidies such as Down's syndrome [1,6,11,12].

**Figure 1.**
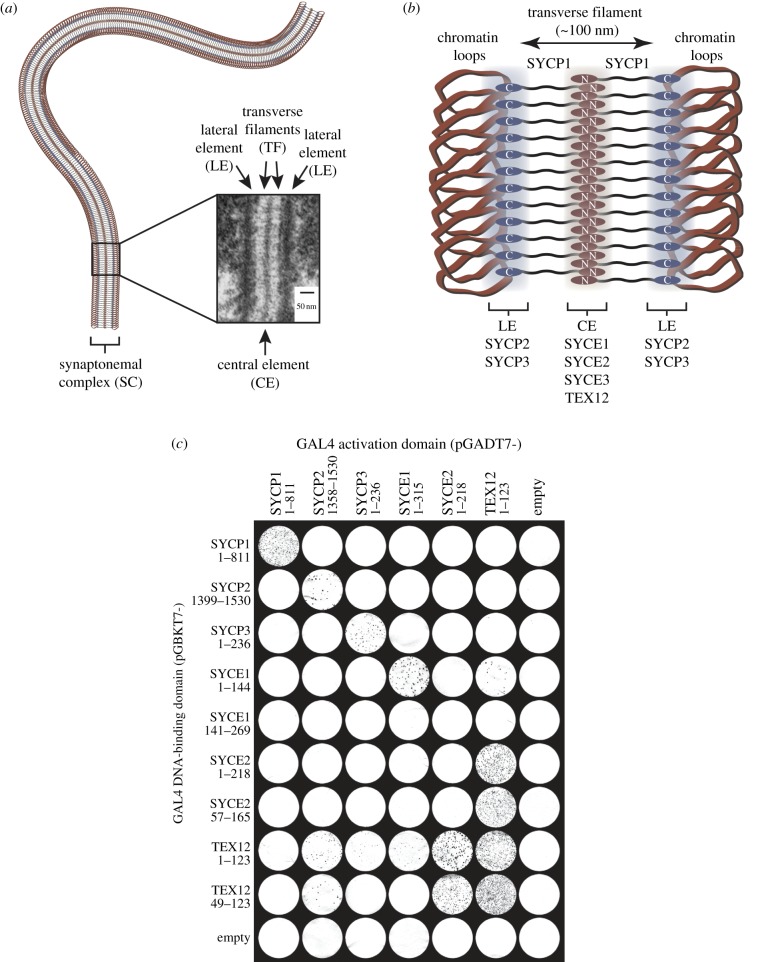
Physical features and protein constituents of the synaptonemal complex. (*a*) Schematic of a synapsed homologous chromosome pair, with electron micrograph of the mouse synaptonemal complex in which central element (CE), lateral element (LE) and transverse filaments (TF) are labelled. The inset electron micrograph image is reproduced from Kouznetsova *et al.* [10] under the Creative Commons Attribution Licence. (*b*) Schematic of the mammalian synaptonemal complex; SYCP1 molecules are orientated according to current models with N-terminal regions in the CE, C-terminal regions in the LE and central regions forming the TF. The LE contains SYCP2 and SYCP3, whereas the CE contains SYCE1, SYCE2, SYCE3 and TEX12. (*c*) Yeast two-hybrid (Y2H) analysis of human SC protein interactions. Y187[pGBKT7-bait] strains were mated with Y2HGold[pGADT7-target] strains, plated on SD/-Ade/-His/-Leu/-Trp/Aba/X-α-Gal plates and then transferred to filters for visualization. Positive reactions depend on activation of the four independent reporter genes: *ADE1*, *HIS3*, *AUR-1C* and *MEL1*. These data are representative of three repeats.

Initially discovered in crayfish spermatocytes [13], the SC has since been observed in a wide range of sexually reproducing organisms, from humans to yeast [14,15]. In all cases, it adopts a remarkably conserved tripartite ribbon-like structure that holds homologous chromosomes together along their entire length. This tripartite structure consists of lateral elements (LEs) running along each chromosome axis, a central element (CE) along the midline and an array of juxtaposed transverse filaments (TFs) that bridge between LEs by interdigitating—much like the teeth of the ‘zipper’—within the CE [2,16–18] (figure 1*a*). In addition to the overall structure, the dimensions of the SC are also well conserved: the central region (comprising TFs and CEs) typically spans 100 nm, whereas LEs and CEs have widths of approximately 50 and 20–40 nm, respectively [14,15].

Assembly and disassembly of the SC are timely events within meiotic prophase I. SC assembly follows the induction of 200–400 double-strand breaks (DSBs) per cell that, through homology searching, establish local alignments between homologous chromosomes [3,6,17,18]. Short tracts of LEs begin to form along chromosome arms and are brought into 400 nm apposition at local alignments. Synapsis of homologous chromosomes nucleates at these sites by bringing LEs into 100 nm apposition and is extended by growth of the CE and TF array along the chromosome axis. SC assembly thus converts local alignments into fully synapsed homologous chromosome pairs. Its three-dimensional architecture further provides the necessary structural framework for completion of meiotic recombination, resulting in DSB resolution and crossover formation [11]. Once accomplished, the SC is disassembled, leaving crossovers as the sole physical links between homologous chromosomes during metaphase I [1–3].

Over the past two decades, seven essential protein constituents of the mammalian SC have been identified [19–25]; all contain predicted α-helical structure, and some contain putative coiled-coils. On the basis of immunofluorescence and immunogold electron microscopy studies, a rudimentary protein map of the SC has been formulated (figure 1*b*). TFs are formed by SYCP1, an elongated protein containing a large central region of predicted coiled-coil with flanking N- and C-terminal domains [21,26]. The N-terminal domain is located within the CE, wherein it is closely associated with CE proteins SYCE1, SYCE2, SYCE3 and TEX12 [23–27], whereas the C-terminal domain localizes to the LE, wherein it contacts LE proteins SYCP2 and SYCP3 [16,22,26–28]. Deficiency of each known SC protein abrogates synapsis, DSB resolution and crossover formation, resulting in complete male/female infertility for SYCP1 and CE proteins, and a sexual dimorphism of male infertility and female subfertility for LE proteins [6–9,25,29,30].

An apparent dichotomy has emerged between CE proteins. SYCE1 and SYCE3 co-localize in a continuous pattern identical to that of SYCP1, and their disruption leads to complete failure of tripartite structure formation [23–25]. By contrast, SYCE2 and TEX12 co-localize in a distinct punctate pattern (although this may reflect antibody properties rather than the underlying protein distribution) and their disruption leads to synaptic failure, albeit with the presence of short stretches of close associations that contain CE-like structure [7,9,23]. Furthermore, SYCE2 and TEX12 co-immunoprecipitate from mouse testis lysate [23]. These findings have led to the suggestion that SYCE1 and SYCE3 function in the initiation of synapsis, whereas SYCE2 and TEX12 function in its extension [4,7,9,25].

Since its discovery over 60 years ago and the recognition of its critical role in meiosis, the accumulating wealth of biological evidence has led to tentative models of SC assembly and disassembly [31–35], and to suggestions of functional roles in mediating recombination, crossover formation and late interference [8,18,36,37]. However, the absence of any detailed biochemical and structural information about the SC and the physical organization of its constituent proteins hampers rational attempts to test current models of SC function, and consequently our understanding of its role in meiosis remains rudimentary. In order to provide a molecular basis of SC function, we have embarked upon the biochemical and structural characterization of purified, recombinant SC proteins.

Here, we describe the reconstitution and biophysical characterization of a stable, constitutive complex between human CE proteins SYCE2 and TEX12. The first biochemical and structural analysis of an essential SC protein component provides molecular insight into assembly of the human SC.

## 3. Material and methods

### 3.1. Yeast two-hybrid

Sequences corresponding to human SYCP1 (1–811), SYCP2 (1399–1530 and 1358–1530), SYCP3 (1–236), SYCE1 (1–315, 1–144 and 141–269), SYCE2 (1–218 and 57–165) and TEX12 (1–123 and 49–123) were cloned into pGBKT7 and pGADT7 vectors (Clontech). Yeast two-hybrid (Y2H) analysis was performed using the Matchmaker Gold Y2H system (Clontech), with protocols based on the manufacturer's instructions. pGBKT7 and pGADT7 vectors were transformed into yeast strains Y187 and Y2H Gold, respectively, according to a standard PEG/ssDNA/LiAc procedure. Y187[pGBKT7-bait] strains were mated with Y2H Gold[pGADT7-target] strains by mixing single colonies of each in 0.5 ml 2xYPDA and incubating at 30°C, 50 r.p.m. for 24 h. Cultures were then diluted 1 in 10 using 0.5xYPDA; 100 µl was plated onto SD/-Leu/-Trp to select for mated colonies, and a further 100 µl was plated onto SD/-Ade/-His/-Leu/-Trp containing aureobasidin A (AbA) and X-α-Gal to select for mated colonies with activation of *ADE1*, *HIS3*, *AUR-1C* and *MEL1* reporter genes. Plates were incubated at 30°C for 5 days. Colonies were lifted onto filters (Whatman No. 5, 70 mm) that were dried, scanned and displayed aligned against a black background.

### 3.2. Recombinant protein expression

For co-expression, sequences corresponding to human SYCE2 (1–218, 57–165, 57–88 and 88–165) with N-terminal MBP-tag and TEX12 (1–123, 24–123, 45–123, 49–123 and 87–123) or SYCE2 (1–218) with N-terminal His-tag (both linkers containing tobacco etch virus (TEV) protease cleavage sequences) were cloned into the two open reading frames of pRSFDuet-1 (Novagen). For separate expression, sequences corresponding to SYCE2 (1–218, 57–165, 57–88 and 88–165) or TEX12 (1–123, 24–123, 45–123, 49–123 and 87–123) with N-terminal MBP- or His-tags were cloned into pMAT11 and pHAT4 vectors, respectively [38]. All constructs were expressed in Rosetta 2 (DE3) cells (Novagen), in 2xYT media, induced with 0.5 mM IPTG for 16 h at 25°C. In the text, usage of the protein names, SYCE2 and TEX12, relates to the full-length sequences, unless stated otherwise, in which case construct boundaries are provided in subscript.

### 3.3. Purification of SYCE2–TEX12 protein complexes

MBP–SYCE2_57−165_ was co-expressed with His–TEX12 or His–TEX12_49–123_ (described earlier). Fusion protein complexes were co-purified from clarified lysate by sequential affinity chromatography using Ni–NTA resin (Qiagen) and amylose resin (NEB); cleaved protein complexes were eluted from the latter column through incubation with TEV protease (Invitrogen). Further purification was achieved through anion-exchange chromatography using a Resource Q 6 ml column (GE Healthcare). Protein complexes were eluted from the Resource Q column in 20 mM Tris pH 8.0, 145 mM KCl, 2 mM DTT, at concentrations of 2–5 mg ml^−1^. All biophysical assays were performed using freshly prepared material. Protein samples were analysed by SDS–PAGE using the NuPAGE Novex Bis–Tris system (Invitrogen), with Coomassie staining performed using SimplyBlue SafeStain (Invitrogen). Densitometry was performed using ImageJ [39]. Protein concentrations were determined by UV spectrophotometry (Varian Cary 50 spectrophotometer), with extinction coefficients and molecular weights calculated by ProtParam (http://web.expasy.org/protparam/). Edman degradation analysis of SYCE2_57–165_–TEX12 solution samples was performed by the Protein and Nucleic Acid Facility (Department of Biochemistry, University of Cambridge).

### 3.4. Circular dichroism spectroscopy

Circular dichroism (CD) data were collected using an Aviv 410 spectropolarimeter (Biophysics facility, Department of Biochemistry, University of Cambridge). Protein complexes SYCE2_57–165_–TEX12 and SYCE2_57–165_–TEX12_49–123_ were analysed at 0.20 and 0.22 mg ml^−1^, respectively, in 10 mM NaH_2_PO_4_ pH 7.5, 150 mM NaF, using a 1 mm path-length quartz cuvette, with 1 nm slit width and 1 s averaging time. CD spectra were recorded at 4°C (between 260 and 185 nm) with 0.5 nm increments; for each sample, raw data from three measurements were averaged, corrected for buffer signal, smoothed and then converted into mean residue ellipticity ([**θ**]). Deconvolution was performed using the CDSSTR algorithm [40] on the DichroWeb server (http://dichroweb.cryst.bbk.ac.uk) [41]. CD temperature melt data were recorded at 222 nm, for 1°C increments between 5°C and 95°C, with 1°C per minute ramping rate, 0.5°C deadband, 30 s incubation time, 1 nm slit width and 1 s averaging time. Raw data were converted to mean residue ellipticity ([**θ**]_222_) using standard equations.

### 3.5. Analytical ultracentrifugation

Sedimentation velocity experiments were performed using a Beckman XL-A analytical ultracentrifuge (Biophysics facility, Department of Biochemistry, University of Cambridge). Protein complexes SYCE2_57–165_–TEX12 and SYCE2_57–165_–TEX12_49–123_ were analysed at 57 and 289 µM, respectively, in 20 mM Tris pH 8.0, 145 mM KCl, 2 mM DTT. Sedimentation velocity experiments were performed at 30 000 r.p.m, 20°C, with absorbance data at 285 nm recorded across cell radii at 0.003 cm intervals, at 3.2 min time intervals, over a total period of 320 min. Protein and buffer parameters were calculated using SEDNTERP, and data were analysed through direct boundary modelling to a continuous c(S) distribution of Lamm equation solutions using SEDFIT [42].

### 3.6. Size-exclusion chromatography–multi-angle light scattering

Absolute molar masses of proteins were determined through size-exclusion chromatography multi-angle light scattering (SEC–MALS). Protein samples (100 µl; 1–5 mg ml^−1^) were loaded onto a Superdex 200 10/300 GL SEC column (GE Healthcare) in 20 mM Tris pH 8.0, 150 mM KCl, 2 mM DTT, at 0.5 ml min^−1^ using an ÄKTA Purifier (GE Healthcare). The column output was fed into a DAWN HELEOS II MALS detector (Wyatt Technology), in which light scattered from a polarized laser source of 664 nm is detected by eight fixed angle detectors, followed by an Optilab T-rEX differential refractometer (Wyatt Technology), which measures absolute and differential refractive index using a 664 nm LED light source at 25°C. Data were collected and analysed using astra 6 software (Wyatt Technology). Molecular masses were calculated across eluted protein peaks through extrapolation from Zimm plots using a d*n*/d*c* value of 0.1850 ml g^−1^; quoted molecular weights and estimated errors relate to the overall mass calculation across a single peak.

### 3.7. Amylose affinity pulldown assay

MBP-fusion SYCE2 constructs were co-expressed with His-tagged TEX12 or SYCE2 constructs (described earlier). For each condition, 1 l cultures were grown, and cells were resuspended in 25 ml of 20 mM Tris pH 8.0, 500 mM KCl, 2 mM DTT, lysed by sonication, clarified by high-speed centrifugation and incubated with 4 ml of amylose resin (NEB) for 1 h at 4°C. After thorough washing, bound complexes were eluted in 10 ml of 20 mM Tris pH 8.0, 150 mM KCl, 30 mM d-maltose, 2 mM DTT. Total protein concentrations were equalized to 3 mg ml^−1^ through dilution or concentration (Millipore Amicon Ultra-4) as appropriate, and analysed by SDS–PAGE (described earlier). This purification method was also used in the preparation of individually expressed MBP–SYCE2 and MBP–TEX12 fusion proteins for analysis by SEC–MALS.

### 3.8. Electron microscopy

Electron microscopy analysis was performed using an FEI Philips CM100 transmission electron microscope (Multi Imaging Unit, University of Cambridge). Protein samples at 100 µM were applied to transmission electron microscopy carbon-coated grids, and negative staining was performing using 0.1 per cent (v/v) uranyl acetate.

### 3.9. Protein sequences and analysis

Protein sequences were extracted from UniProtKB; multiple sequence alignments were performed using MUSCLE (EBI) and were displayed using Jalview v. 2.0 (www.jalview.org) [43]. Secondary structure predictions were performed using Jnet (http://www.compbio.dundee.ac.uk/www-jpred/), PsiPred v. 3.0 (http://bioinf.cs.ucl.ac.uk/psipred/), Porter (http://distill.ucd.ie/porter/) and Sopma (http://npsa-pbil.ibcp.fr/cgi-bin/npsa_automat.pl?page=npsa_sopma.html).

## 4. Results

### 4.1. Identification of a constitutive SYCE2–TEX12 complex

Given the apparent intricacy of the molecular architecture of the SC, we reasoned that SC proteins might exist in constitutive multi-component complexes. We thus set out to identify interactions between human SC proteins that would facilitate their biochemical and structural analysis. This was achieved by a yeast Y2H grid screen of human SC components using the Matchmaker Gold Y2H system, in which positive interactions are determined by the activation of the four independent reporter genes *ADE1*, *HIS3*, *AUR-1C* and *MEL1*. This revealed self-association of SYCP1, SYCP2, SYCP3, SYCE1 and TEX12, consistent with previous reports [21,23,24,30,32], and a robust interaction between CE proteins SYCE2 and TEX12 that was detected in both directions (figure 1*c*). We did not identify other heterotypic SC protein interactions. This may be due to their non-binary nature, the high stringency nature of this screen (designed to identify only strong interactions), or steric interference of the Y2H fusion proteins. Complex formation between SYCE2 and TEX12 is entirely consistent with previous reports of their co-localization, co-immunoprecipitation and the phenotypic similarity of their individual knockouts [7,9,23]. Accordingly, we decided to focus our efforts on the putative SYCE2–TEX12 interaction.

**Figure 2.**
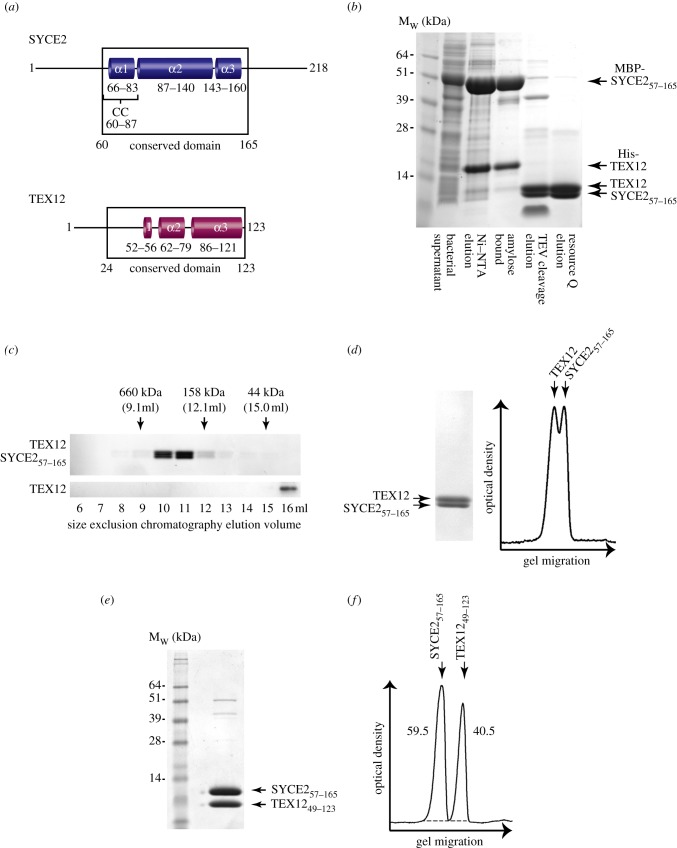
Identification of a constitutive equimolar complex between central element proteins SYCE2 and TEX12. (*a*) Schematic of human SYCE2 and TEX12 protein sequences. The central region of SYCE2 (residues 60–165) shows evolutionary conservation; α-helical structure is predicted for residues 66–83 (α1), 87–140 (α2) and 143–160 (α3), and the coiled-coil (CC) formation is predicted for residues 60–87. The central and C-terminal region of TEX12 (residues 24–123) show evolutionary conservation; α-helical structure is predicted for residues 52–56 (α1), 62–79 (α2) and 86–121 (α3). For full sequence alignments, secondary structure and coiled-coil predictions, see electronic supplementary material, figures S1 and S2. (*b*) Coomassie-stained SDS–PAGE showing co-expression in bacteria and co-purification of the SYCE2_57–165_–TEX12 complex by Ni–NTA affinity chromatography, amylose affinity chromatography, TEV cleavage and anion-exchange chromatography. (*c*) Coomassie-stained SDS–PAGE showing size-exclusion chromatography analysis of SYCE2_57–165_–TEX12 in comparison with His-TEX12; elution positions of gel filtration standards are shown. (*d*) Densitometry analysis of purified SYCE2_57–165_–TEX12; for analysis, the sample was diluted until peaks for constituent proteins became clearly defined, as shown. (*e*) Coomassie-stained SDS–PAGE of SYCE2_57–165_–TEX12_49–123_; this complex was purified in an identical manner to SYCE2_57–165_–TEX12. (*f*) Densitometry analysis of SYCE2_57–165_–TEX12_49–123_; integrated intensities of SYCE2_57–165_ and TEX12_49–123_ peaks account for 59.5% and 40.5% of the total signal, closely matching their theoretical equimolar mass percentages of 59.1% and 40.9%, respectively.

Sequence analysis of SYCE2 reveals that this 218 amino acid protein consists of a central evolutionarily conserved domain of three predicted α-helices, the first of which forms a putative coiled-coil (at confidence level greater than 90%), flanked by divergent, unstructured N- and C-terminal extensions (see figure 2*a*; electronic supplementary material, figure S1). TEX12 is a highly conserved 123 amino acid protein, containing three predicted α-helices in its central and C-terminal regions, with a divergent N-terminus (see figure 2*a*; electronic supplementary material, figure S2). Expression and purification of individual SYCE2 and TEX12 only allowed for recovery of small amounts of material after removal of affinity tags, which was unsuitable for biophysical analysis. By contrast, SYCE2 and TEX12 co-expression conferred a large increase in the solubility and stability of both protein components. In the case of the full-length protein complex, removal of affinity tags revealed considerable degradation of SYCE2. As the N- and C-terminal extensions of SYCE2 are divergent or absent in other species (see electronic supplementary material, figure S1), and are dispensable for interaction with TEX12 (figure 1*c*), we co-expressed TEX12 with the central conserved region of SYCE2, spanning residues 57–165. This eliminated degradation, enabling the purification of an SYCE2–TEX12 complex suitable for biophysical analysis. Co-purification of SYCE2_57–165_ and TEX12 over three distinct chromatography steps (figure 2*b*) and further co-elution in size-exclusion chromatography (figure 2*c*) confirmed the presence of a strong association between SYCE2 and TEX12. Indeed, we could not identify a non-denaturing biochemical condition in which the SYCE2–TEX12 complex is disrupted. We thus conclude that their interaction is both highly stable and constitutive. Furthermore, SDS–PAGE band densitometry (figure 2*d*) and Edman degradation analysis (data not shown) of the purified SYCE2_57–165_–TEX12 complex indicate that it is equimolar.

We further prepared an SYCE2_57–165_–TEX12_49–123_ complex in which the natively unstructured N-terminal region of TEX12 that is dispensable for interaction with SYCE2 (figure 1*c*) was deleted. The SYCE2_57–165_–TEX12_49–123_ complex was purified in an identical manner to SYCE2_57–165_–TEX12, showed comparable stability (figure 2*e*) and was confirmed to be equimolar through Coomassie-stained SDS–PAGE band densitometry (figure 2*f*). Our biochemical analysis thus confirms that CE proteins SYCE2 and TEX12 form a constitutive equimolar complex.

### 4.2. High helical content and thermal stability of the SYCE2–TEX12 complex

**Figure 3.**
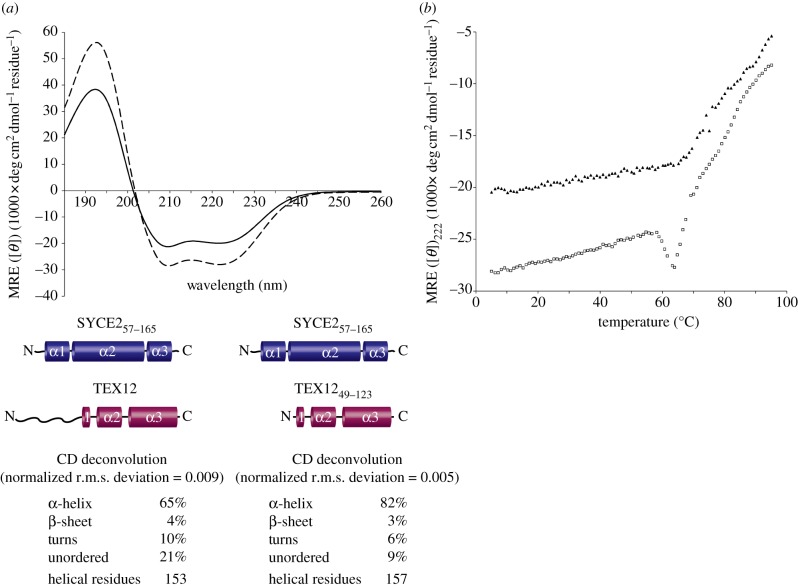
CD analysis of SYCE2–TEX12 reveals high helical content and thermal stability. (*a*) Far UV CD spectra of SYCE2_57–165_–TEX12 (solid line) and SYCE2_57–165_–TEX12_49–123_ (dashed line) recorded between 260 and 185 nm in 0.5 nm increments, in mean residue ellipticity, MRE ([**θ**]) (1000× deg cm^2^ dmol^−1^ residue^−1^). Data were deconvoluted using the CDSSTR algorithm and data fitted at normalized r.m.s. deviation values of 0.009 and 0.005, respectively. SYCE2_57–165_–TEX12 contains 65% helical content (153 helical residues), 4% β-sheet, 10% turns and 21% unordered elements. SYCE2_57–165_–TEX12_49–123_ contains 82% helical content (157 helical residues), 3% β-sheet, 6% turns and 9% unordered elements. For comparison, predicted helical contents are 64% and 79%, respectively (150 predicted helical residues for both constructs). (*b*) Temperature melts of SYCE2_57–165_–TEX12 (triangles) and SYCE2_57–165_–TEX12_49–123_ (squares), recording the CD helical signature at 222 nm in MRE ([**θ**]_222_) at 1°C intervals between 5°C and 95°C.

As the first stage of structural characterization, we assessed secondary structure composition of SYCE2–TEX12 by CD spectroscopy (figure 3*a*). Far UV spectra of SYCE2_57–165_–TEX12 showed the presence of 65 per cent α-helical content (153 helical residues), remarkably close to its predicted α-helical content of 64 per cent (150 helical residues). CD analysis of the SYCE2_57–165_–TEX12_49–123_ complex showed an increase in relative α-helical content to 82 per cent (157 helical residues) with concomitant reduction in unordered signal. These data confirm that the N-terminal region of TEX12 is unstructured, validating our subsequent use of SYCE2_57–165_–TEX12_49–123_ in structural analysis, and demonstrate high helical content within the central region of SYCE2 and the central and C-terminal regions of TEX12.

We assessed the thermal stability of the SYCE2–TEX12 complex by measuring the α-helical signature ellipticity at 222 nm over the temperature range 5–95°C (figure 3*b*). SYCE2_57–165_–TEX12 showed a reversible linear decline in ellipticity (i.e. typical of α-helical fraying [44]) up to 65°C, with irreversible cooperative unfolding beyond this point. Similar data were obtained for SYCE2_57–165_–TEX12_49–123_, albeit with irreversible conformation change and subsequent unfolding occurring at the slightly lower temperature of 55°C. The considerable resistance to thermal denaturation confirmed the high conformational stability of the SYCE2–TEX12 complex.

### 4.3. The SYCE2–TEX12 complex is a hetero-octamer

**Figure 4.**
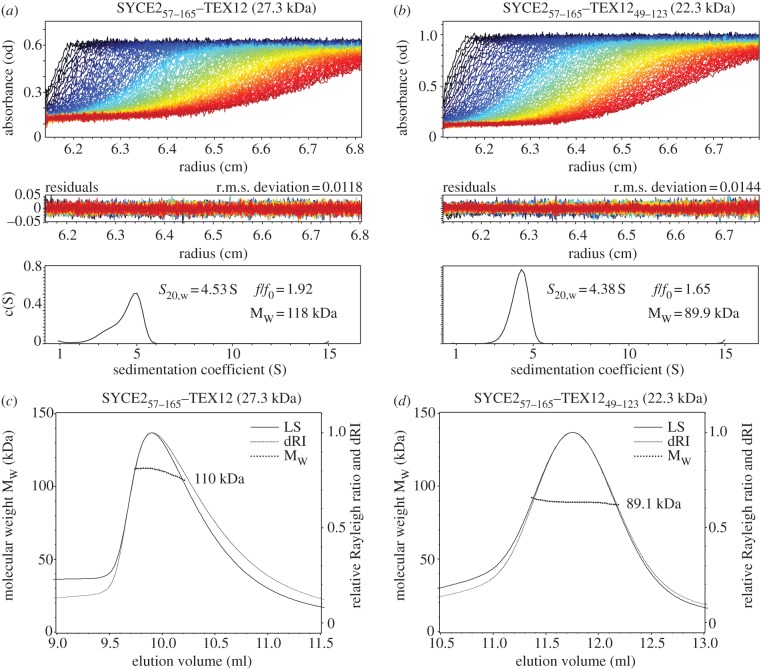
The SYCE2–TEX12 complex is a hetero-octamer. (*a*,*b*) Analytical ultracentrifugation (AUC) sedimentation velocity analysis of SYCE2–TEX12 protein complexes: fringes collected by absorbance measurements at 285 nm (top panels), residuals from Lamm equation solutions (middle panels) and resultant continuous c(S) distributions in the range 1–15 S (bottom panels). (*a*) SYCE2_57–165_–TEX12 data were fitted with a r.m.s. deviation of 0.0118, showing a mean sedimentation coefficient of 4.53 S, best fit frictional ratio (*f*/*f*_0_) of 1.92 and calculated mean molecular weight of 118 kDa. Interpreted species account for 77.5% of total. Its theoretical 4 : 4 size is 109 kDa. (*b*) SYCE2_57–165_–TEX12_49–123_ data were fitted with a r.m.s. deviation of 0.0144, showing a mean sedimentation coefficient of 4.38 S, best fit frictional ratio (*f*/*f*_0_) of 1.65 and calculated mean molecular weight of 89.9 kDa. Interpreted species account for 84.9% of total. Its theoretical 4 : 4 size is 89.0 kDa. (*c*,*d*) SEC–MALS analysis of SYCE2–TEX12 protein complexes; light scattering (LS) and differential refractive index (dRI) are plotted alongside fitted molecular weights (M_w_). (*c*) SYCE2_57–165_–TEX12 eluted in a majority (approx. 70%) single peak corresponding to a species of 110 kDa (±0.080%) with polydispersity of 1.001 (±0.113%). The remainder constituted high molecular weight aggregates. (*d*) SYCE2_57–165_–TEX12_49–123_ eluted in a single peak, corresponding to a species of 89.1 kDa (±0.176%) with polydispersity of 1.000 (±0.248%).

We next set out to determine the oligomeric status of the SYCE2–TEX12 complex. Analytical ultracentrifugation (AUC) sedimentation velocity data for SYCE2_57–165_–TEX12 were fitted to a continuous c(S) distribution, resulting in a single skewed peak of sedimentation coefficient 4.53 S, with fitted frictional ratio 1.92 and estimated molecular weight 118 kDa (figure 4*a*). As we have previously determined the complex to be equimolar, the AUC analysis is most consistent with a hetero-octameric assembly formed by four chains each of SYCE2 and TEX12, corresponding to a theoretical molecular weight of 109 kDa. The skewed peak and slight disparity between estimated and theoretical molecular weights are likely due to the unstructured N-terminal region of TEX12. AUC analysis of SYCE2_57–165_–TEX12_49–123_ showed a single symmetrical peak of 4.38 S, with fitted frictional ratio 1.65 and estimated molecular weight 89.9 kDa (figure 4*b*), closely matching an equimolar hetero-octamer size of 89.0 kDa. Reduction in frictional ratio confirms the flexible unstructured nature of the TEX12 N-terminus, and a frictional ratio of 1.65 for SYCE2_57–165_–TEX12_49–123_ indicates significant asymmetry within this central core, suggesting that the complex adopts an extended rather than a globular conformation.

To confirm the size of the SYCE2–TEX12 complex, we employed SEC–MALS, in which native molecular weights are determined absolutely, overcoming the ambiguity of frictional ratio fitting in AUC. SYCE2_57–165_–TEX12 eluted in a majority peak of molecular weight 110 kDa (figure 4*c*), with some high molecular weight aggregation, whereas SYCE2_57–165_–TEX12_49–123_ eluted in a single peak of molecular weight 89 kDa (figure 4*d*). Thus, the molecular weights determined by SEC–MALS match closely the theoretical sizes of 109 and 89 kDa for equimolar hetero-octameric assemblies of SYCE2_57–165_–TEX12 and SYCE2_57–165_–TEX12_49–123_, respectively.

### 4.4. SYCE2 is a constitutive tetramer that multimerizes via its central α2–3 region

**Figure 5.**
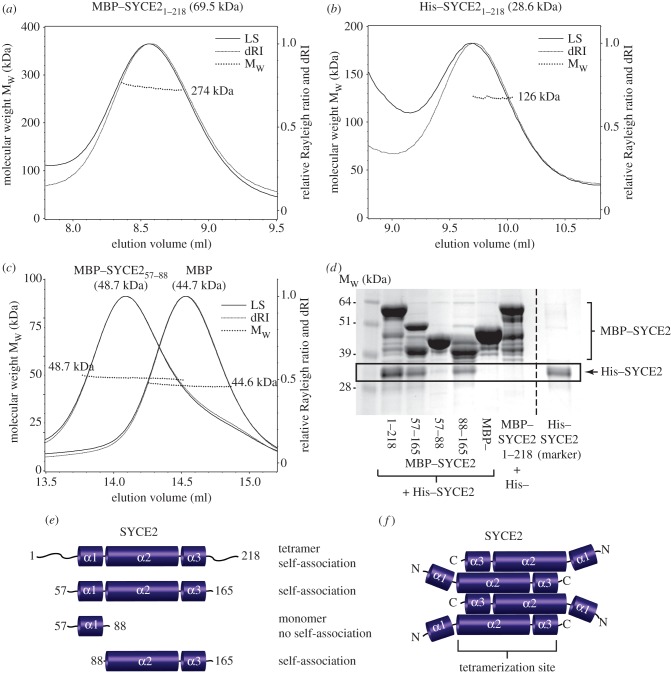
SYCE2 undergoes tetramerization through its central α2–3 region. (*a–c*) SEC–MALS analysis of MBP–SYCE2 fusion proteins; light scattering (LS) and differential refractive index (dRI) are plotted alongside fitted molecular weights (M_w_). (*a*) MBP–SYCE2 eluted in a single peak of 274 kDa (±0.065%) with polydispersity of 1.000 (±0.092%); its theoretical tetramer size is 278 kDa. (*b*) His–SYCE2 eluted in approximately equal quantities in a single peak and in higher molecular weight aggregates within the void volume; the single peak corresponds to 126 kDa (±0.473%) with polydispersity of 1.000 (±0.669%). Its theoretical tetramer size is 115 kDa. (*c*) MBP–SYCE2_57–88_ and MBP eluted in single peaks of 48.7 kDa (±0.131%, polydispersity of 1.000 ± 0.184%) and 44.6 kDa (±0.077%, polydispersity of 1.000 ± 0.108%), respectively. Their theoretical monomer sizes are 48.7 and 44.7 kDa. (*d*) Amylose pull-down of His–SYCE2 following its co-expression with MBP–SYCE2 1–218, 57–165, 57–88 and 88–165, and with free MBP, visualized by Coomassie staining. Purified His–SYCE2 is included, as a marker, in the lane on the right of the broken line. (*e*) Summary of data: tetramer formation is demonstrated for full-length SYCE2, and monomer formation for its N-terminal α1 region (amino acids 57–88); self-association is demonstrated for full-length, central α1–3 (amino acids 57–165) and α2–3 (amino acids 88–165) regions of SYCE2. (*f*) Model for SYCE2 in which tetramerization is mediated by the central α2–3 region.

The realization that interaction of SYCE2 and TEX12 leads to an octameric assembly raises the question of their oligomeric status in the absence of the protein partner. As production of isolated recombinant SYCE2 and TEX12 proved difficult, we resorted to the use of MBP-fusion tags in order to improve solubility and stability. SEC–MALS analysis of MBP–SYCE2 fusion protein (see figure 5*a*; electronic supplementary material, figure S3*a*) revealed a single peak of molecular weight 274 kDa, consistent with an MBP–SYCE2 tetramer of theoretical molecular weight 278 kDa. These findings were confirmed by a SEC–MALS analysis of His-tagged SYCE2 (see figure 5*b*; electronic supplementary material, figure S3*b*) that, despite significant instability and aggregation of the sample, indicated a molecular weight of 126 kDa, against a theoretical tetramer size of 115 kDa. We thus conclude that SYCE2 exists as a tetramer in solution. We note that SYCE2 self-association was not detected by Y2H (figure 1*c*); this may be due to the lack of dynamic exchange between the two populations of SYCE2 complexes upon yeast mating, or steric interference of Y2H fusion proteins.

To explore the molecular determinants of SYCE2 tetramerization, we dissected the SYCE2 sequence on the basis of the three predicted α-helices within its central evolutionarily conserved domain. As an MBP-fusion protein, the α1 region of SYCE2 (amino acids 57–88) proved highly stable, and was determined by SEC–MALS to have a molecular weight of 48.7 kDa (figure 5*c*; electronic supplementary material, figure S3*c*,*d*), precisely matching its theoretical monomer size. We could not obtain SEC–MALS data for MBP-fusion proteins corresponding to the α1–3 (amino acids 57–165) and α2–3 (amino acids 88–65) regions of SYCE2, presumably owing to their instability in the absence of TEX12 (data not shown). To overcome this, we assessed the ability of MBP–SYCE2 fusion constructs to self-associate with His–SYCE2 by amylose pull-down following co-expression in bacteria (figure 5*d*). Pull-down experiments revealed His–SYCE2 binding to full-length, α1–3 and α2–3 regions of SYCE2, but not to its α1 region. These data confirm self-association of full-length and central α1–3 region of SYCE2, and further demonstrate that while the N-terminal α1 region is monomeric, the central α2–3 region is sufficient for oligomerization (figure 5*e,f*).

### 4.5. TEX12 is a constitutive dimer that self-associates via its central α1–2 region

**Figure 6.**
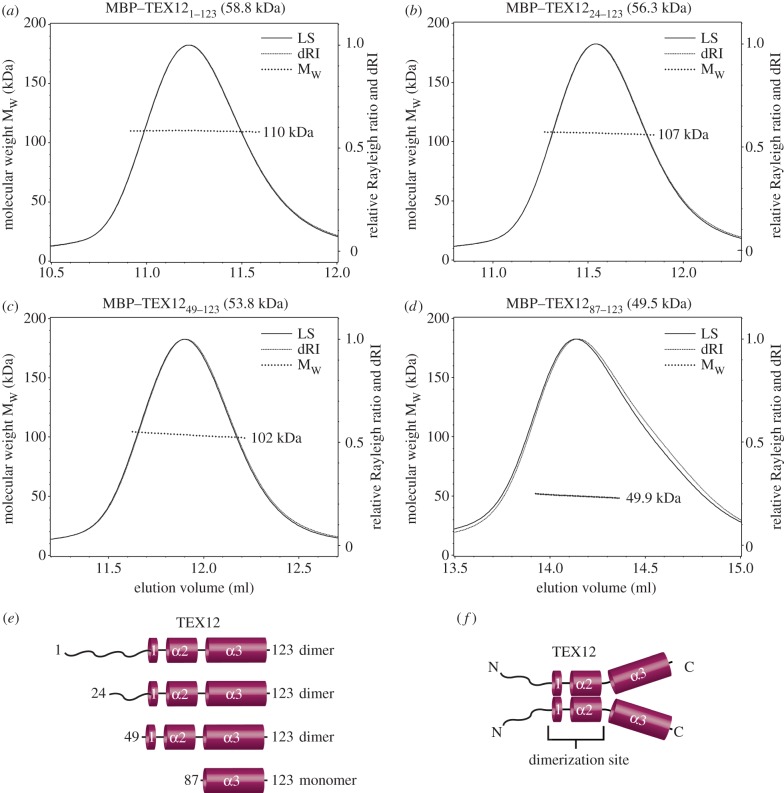
TEX12 undergoes dimerization through its central α1–2 region. (*a*–*d*) SEC–MALS analysis of MBP–TEX12 fusion proteins in which the N-terminus of TEX12 is progressively truncated; light scattering (LS) and differential refractive index (dRI) are plotted alongside fitted molecular weights (M_w_). All proteins eluted in single peaks. (*a*) MBP–TEX12_1–123_ is 110 kDa (±0.110%), with polydispersity of 1.000 (±0.155%); its theoretical dimer size is 118 kDa. (*b*) MBP-TEX12_24–123_ is 107 kDa (±0.203%), with polydispersity of 1.000 (±0.286%); its theoretical dimer size is 113 kDa. (*c*) MBP-TEX12_49–123_ is 102 kDa (±0.095%), with polydispersity of 1.000 (±0.133%); its theoretical dimer size is 108 kDa. (*d*) MBP-TEX12_87–123_ is 49.9 kDa (±0.102%), with polydispersity of 1.000 (±0.144%); its theoretical monomer size is 49.5 kDa. (*e*) Summary of data: dimer formation is maintained in all N-terminal truncations other than 87–123. (*f*) Model for TEX12 in which dimerization is mediated by the central α1–2 region.

We next assessed the oligomer status of isolated TEX12. As for SYCE2, it proved necessary to express and purify TEX12 as an MBP-fusion protein (see electronic supplementary material, figure S3*e*) in order to obtain recombinant protein suitable for biophysical analysis. SEC–MALS revealed a single peak of molecular weight 110 kDa (figure 6*a*), consistent with an MBP–TEX12 homodimer (its theoretical homodimer size is 118 kDa). The molecular determinants of dimerization were explored by a SEC–MALS analysis of a series of MBP-fusion proteins in which the N-terminus of TEX12 was progressively truncated (see figure 6*b–d*; electronic supplementary material, figure S3*e*). Dimerization was retained for MBP-fusion constructs TEX12_24–123_ and TEX12_49–123_ that contain the three predicted helices of the evolutionarily conserved domain, α1–3 (observed molecular weights of 107 and 102 kDa, respectively, and theoretical dimer sizes of 113 and 108 kDa). Thus, self-association of TEX12 is maintained in the SYCE2_57–165_–TEX12_49–123_ complex. However, deletion of the α1–2 region abrogated dimerization, as the MBP–TEX12_87–123_ construct containing only the C-terminal α3 region had a molecular weight of 49.9 kDa (theoretical monomer size of 49.5 kDa). We conclude that TEX12 dimerizes via its central α1–2 region (figure 6*e*,*f*).

### 4.6. A molecular model for the SYCE2–TEX12 hetero-octamer

**Figure 7.**
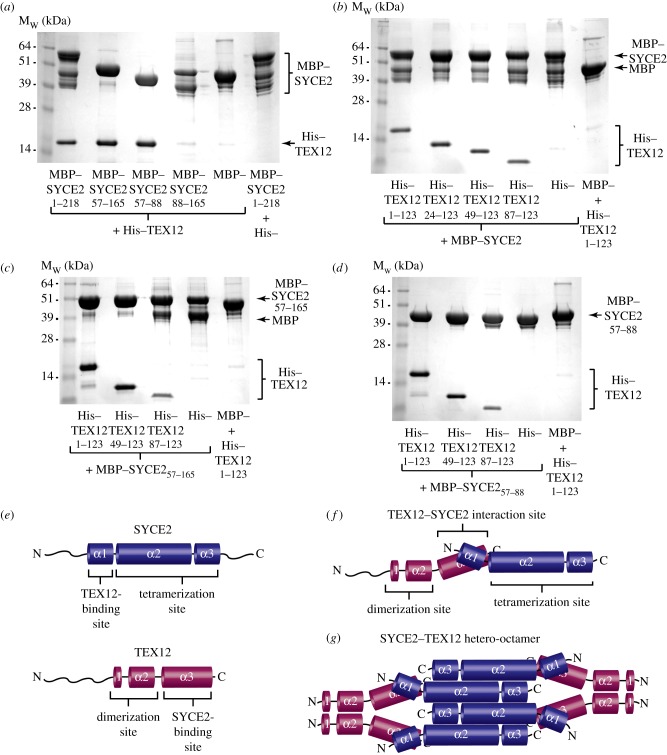
Molecular determinants of the SYCE2–TEX12 interaction. (*a*) Amylose pull-down of His–TEX12 following its co-expression with MBP–SYCE2 1–218, 57–165, 57–88 and 88–165, and with free MBP, visualized by Coomassie staining. Integrated intensities of TEX12 bands were measured as 62% (1–218), 96% (57–88), 5% (88–165) and 3% (free MBP) relative to 57–165. The multiple bands of MBP–SYCE_88–165_ result from its degradation during expression; the ability of this construct to pull-down His–SYCE2 (figure 5*d*) is an important positive control for this assay. (*b–d*) Amylose pull-down of His–TEX12 1–123, 24–123, 49–123 and 87–123 following its co-expression with MBP–SYCE2 and with free MBP, visualized by Coomassie staining. Pull-down reactions were performed using (*b*) MBP–SYCE2_1–218_, (*c*) MBP–SYCE2_57–165_ and (*d*) MBP–SYCE2_57–88_ as the bait protein. (*e*) Schematic showing the location of TEX12-binding and tetramerization sites within α1 and α2–3 regions of SYCE2, and dimerization and SYCE2-binding sites within α1–2 and α3 regions of TEX12. (*f*) Modular structure of SYCE2 and TEX12 proteins. (*g*) Model of the SYCE2–TEX12 hetero-octamer: an SYCE2 tetramer binds two TEX12 dimers through constitutive 1 : 1 interactions between the α1 region of SYCE2 and α3 region of TEX12.

As a further step in the analysis of the SYCE2–TEX12 complex, we investigated the molecular determinants of the SYCE2–TEX12 interaction by amylose pull-down of bacterial extracts containing over-expressed MBP–SYCE2 and His–TEX12 constructs. First, we assessed TEX12 binding by SYCE2 (figure 7*a*). His–TEX12 binding was detected for full-length, α1–3 and α1 regions of SYCE2, but not for its α2–3 region. Thus, the N-terminal α1 region of SYCE2 spanning residues 57–88 is necessary and sufficient for interaction with TEX12. Interestingly, despite being monomeric in solution, SYCE2_57–88_ contains a predicted coiled-coil, suggesting that the SYCE2–TEX12 interaction may take the form of a heterotypic coiled-coil. These data suggest a modular structure for SYCE2, with mutually independent functions pertaining to the N-terminal α1 region that binds TEX12, and the central α2–3 region that is responsible for tetramerization (figure 7*e*).

We next assessed SYCE2-binding by TEX12. Interactions with MBP–SYCE2 and MBP–SYCE2_57–165_ were detected for all N-terminal truncations of TEX12 down to and including its α3 region alone (figure 7*b,c*), indicating that the C-terminal α3 region of TEX12 spanning amino acids 87–123 is necessary and sufficient for interaction with SYCE2. Thus, TEX12 structure contains mutually independent functional modules as observed for SYCE2, with a central α1–2 region that mediates dimerization, and a C-terminal α3 region responsible for SYCE2 binding (figure 7*e*). Interactions with MBP–SYCE2_57–88_ were further detected for all N-terminal truncations of TEX12 down to and including its α3 region alone (figure 7*d*), but not for the N-terminal or α1–2 regions of TEX12 (see electronic supplementary material, figure S4), confirming a direct interaction between the N-terminal α1 region of SYCE2 and the C-terminal α3 region of TEX12. Interestingly, the stabilizing effect conferred by TEX12 onto MBP–SYCE2 (as assessed by proteolytic degradation of the fusion protein) is substantially diminished for the α3 region of TEX12 (figure 7*b*,*c*), suggesting that stabilization is dependent on TEX12 dimerization.

On the basis of these findings, we propose a molecular model for SYCE2–TEX12 hetero-octamer formation in which an SYCE2 tetramer binds two TEX12 dimers through 1 : 1 interactions between N-terminal α1 regions of SYCE2 and C-terminal α3 regions of TEX12 (figure 7*f*,*g*).

### 4.7. Higher-order structure formation of SYCE2–TEX12

**Figure 8.**
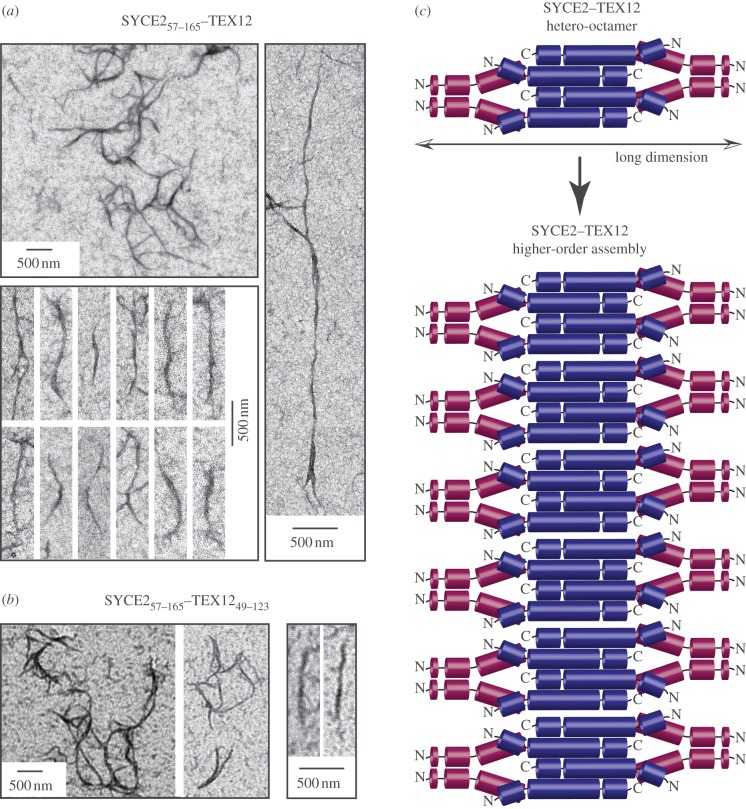
Transmission electron microscopy analysis of SYCE2–TEX12 complexes. (*a*) SYCE2_57–165_–TEX12 forms numerous regular filament-like structures (top-left panel); individual filaments are approximately 40 nm wide, and vary in length from 300 nm to 1 µm (bottom-left panel). Occasional extended filamentous structures of length up to 5 µm are also observed (right panel). (*b*) Similar filament-like structures are observed for SYCE2_57–165_–TEX12_49–123_, shown over wide fields (left panel) and for individual filaments (right panel). (*c*) Model for assembly of SYCE2–TEX12 into higher-order structures through lateral associations of hetero-octamers, with the filament width determined by the long axis. These SYCE2–TEX12 higher-order assemblies may constitute key longitudinal structural components of the SC central element.

The observation that SYCE2 and TEX12 associate constitutively in a hetero-octameric assembly raises the question of the biological role of the SYCE2–TEX12 interaction in SC function. As SYCE2 and TEX12 co-localize to the same molecular network that extends throughout the CE, we decided to investigate whether the SYCE2–TEX12 complex could self-associate in large supramolecular structures of comparable size to the known physical dimensions of the SC. Electron microscopy analysis of SYCE2_57–165_–TEX12 and SYCE2_57–165_–TEX12_49–123_ complexes revealed their concentration-dependent assembly into extended, filamentous structures that are approximately 40 nm wide and range in length from 300 nm to 1 μm (figure 8*a*,*b*). The dimensions of the filaments resemble those of the CE within the SC [14,15], raising the possibility that the SYCE2–TEX12 filaments might represent structural components of the CE. To relate this to our solution studies of SYCE2–TEX12, while the majority species observed were hetero-octamers, a minority of higher-order species were observed, the proportion and size of which were irreversibly increased by protein concentration (data not shown). It is sensible to envisage that assembly of SYCE2–TEX12 complexes into higher-order filamentous structures within the CE might be a dynamic process driven by low-affinity interactions between SYCE2–TEX12 complexes, in contrast to the high-affinity, constitutive interactions that hold together the SYCE2–TEX12 hetero-octamer (figure 8*c*).

## 5. Discussion

Since its discovery in 1956, the tripartite structure of the SC has become recognized as a physical hallmark of meiosis. However, despite its essential role in meiotic cell division, the molecular structure, mechanism of assembly and function of the SC remain largely unknown. One of the principal challenges of studying the SC at the molecular level is the difficulty in producing recombinant versions of the SC proteins, which has precluded so far their biochemical and structural analysis. Here, we have overcome this problem by defining a stable and constitutive complex between CE proteins SYCE2 and TEX12, as an equimolar hetero-octamer, resulting from the constitutive interaction of one SYCE2 tetramer with two TEX12 dimers. The assembly of SYCE2–TEX12 hetero-octamers into higher-order structures suggests a possible architectural role of the complex in CE structure.

The constitutive nature of the SYCE2–TEX12 interaction is consistent with their co-localization pattern and co-immunoprecipitation, as well as the similar phenotype of synaptic failure induced by their individual disruption [7–9,23–25]. It is likely that the SYCE2–TEX12 hetero-octamers form immediately upon expression in meiotic cells and that they constitute the dynamic form that is transported to chromosomes for SC assembly. The realization that SYCE2 and TEX12 associate spontaneously into hetero-oligomers raises the question of whether other SC protein components exist in constitutive complexes. Clear candidates are SYCE1 and SYCE3, which, similar to SYCE2 and TEX12, have a shared functional role and localization pattern within the CE [8,24,25].

A molecular model of the SYCE2–TEX12 hetero-octamer was constructed from biophysical and pull-down analyses of protein truncations (figure 7*f*,*g*). SYCE2 and TEX12 share a modular structure in which both proteins contain distinct self-association and heterotypic interaction sites. SYCE2 undergoes tetramerization through its central α2–3 region, whereas TEX12 dimerizes through its central α1–2 region. Heterotypic association is mediated by the N-terminal α1 region of SYCE2 and the C-terminal α3 region of TEX12, possibly through coiled-coil formation. Thus, assembly of the SYCE2–TEX12 hetero-octamer results from four 1 : 1 interactions between an SYCE2 tetramer and two TEX12 dimers. The strong reciprocal affinity of SYCE2 and TEX12, and high stability of the resulting complex, indicates a large degree of reciprocal stabilization of the two protein partners.

The regular filamentous appearance of the higher-order structures formed by SYCE2–TEX12 complexes that extend to micrometre scale suggest that they might represent ‘bona fide’ architectural components of the CE. Given the high asymmetry of the SYCE2–TEX12 hetero-octamer, we postulate that the long dimension of the complex constitutes the width of the higher-order structures and that formation of extended filaments occurs by lateral associations of hetero-octamers (figure 8*c*). As the large majority of the SYCE2–TEX12 complex exists in solution as individual hetero-octamers, lateral associations are probably low-affinity and dependent on high protein concentrations of the complex. These weak associations between SYCE2–TEX12 hetero-octamers may exert considerable cooperativity, creating a stable higher-order structure. Within the cell, the formation of such structures might be induced by high local concentration of the complex at the developing SC and may be further stabilized by specific interactions with other SC proteins.

To assess the potential role of SYCE2–TEX12 higher-order structures within the CE, we refer to previous electron microscopy three-dimensional reconstruction studies of the SC central region. In insects, the CE has well-defined, ladder-like structures, provided by pairs of stacked pillars orientated perpendicular to the axis, which are connected vertically, transversely and longitudinally by fibrous bridges [45–47]. The mammalian CE is, by contrast, far more amorphous; nevertheless, putative transverse and longitudinal components have been reported [45,46]. The filamentous nature of SYCE2–TEX12 higher-order structures is most consistent with a role as longitudinal CE components that extend synapsis in recurrent discrete steps along the chromosome axis. This is in agreement with the observed failure of extension, but retention of synaptic initiation, upon disruption of SYCE2 or TEX12 in meiotic cells [7,9], and provides molecular explanation for their distinct punctate staining pattern along the length of the SC [23,24].

Our findings can be combined with existing biological data in a model for SC assembly. At sites of initiation, growth of SYCE2–TEX12 filaments may extend the CE, in synchrony with concomitant extension of the SYCP1 TF array. Full synapsis of homologous chromosomes may be achieved through repeated episodes of initiation and extension of SYCE2–TEX12 filaments, resulting in concurrent, reciprocal stabilization of the CE and the flanking arrays of TFs. While it remains unknown how SYCE2–TEX12 complexes associate with TFs, possibilities include direct interactions with SYCP1 or indirect interactions mediated by synaptic initiation proteins such as SYCE1 and SYCE3 [23–25]. To extend the familiar analogy of the SC as a ‘zipper’, if SYCP1 molecules are the teeth, SYCE2–TEX12 seemingly acts as the slider, pulling the teeth together from initiation sites and extending synapsis along the entire chromosome axis.

As a complete catalogue of protein factors important for SC assembly and functions emerges from biological studies, it will become increasingly possible to attempt the partial or complete biochemical reconstitution of the process of SC assembly that takes place during meiosis. An important outcome of this work is the demonstration that biochemical and biophysical studies of SC proteins are both feasible and necessary in order to understand the molecular basis of SC function.

## Supplementary Material

Electronic supplementary material
