# Supplementary material for: Structural analysis of the human SYCE2–TEX12 complex provides molecular insights into synaptonemal complex assembly
Source: Open Biol. 2012 Jul;2(7):120099. doi: 10.1098/rsob.120099 (PMC3411106; doi:10.1098/rsob.120099)
Supplement: Electronic supplementary material [file rsob120099-s1.pdf]

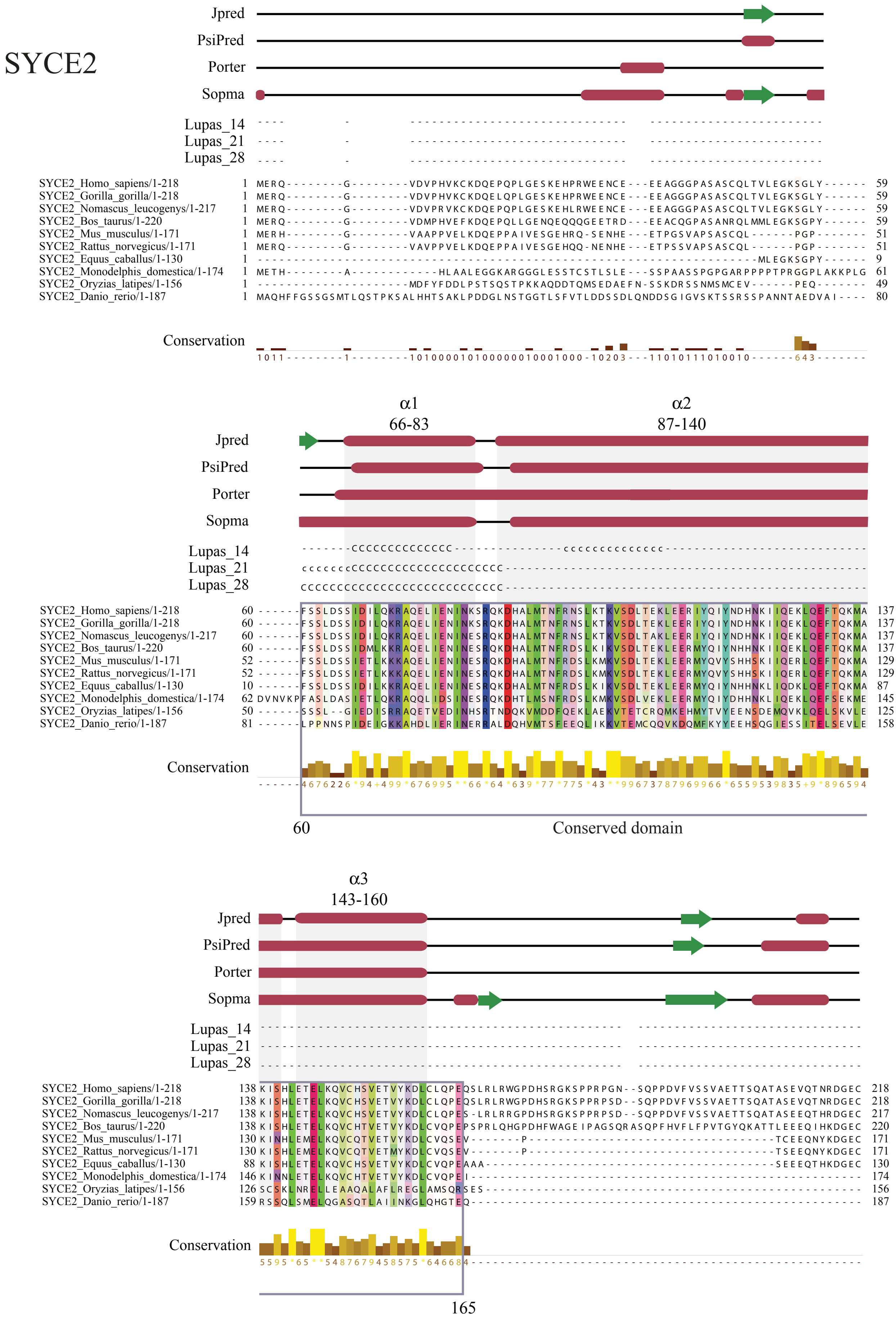


**Figure S1. SYCE2 sequence alignment, including secondary structure and coiled-coil prediction.** Predicted α-helices and β-sheets are shown for four secondary structure prediction algorithms: Jpred, PsiPred, Porter and Sopma. Annotated helices α1, α2 and α3 relate to the Jpred prediction. Coiled-coil prediction (JNet: Lupas algorithm) is shown for windows of 14, 21 and 28 residues, with the probability of coiled-coil formation for each residue indicated by - (<50%) , c (50-90%) or C (>90%).

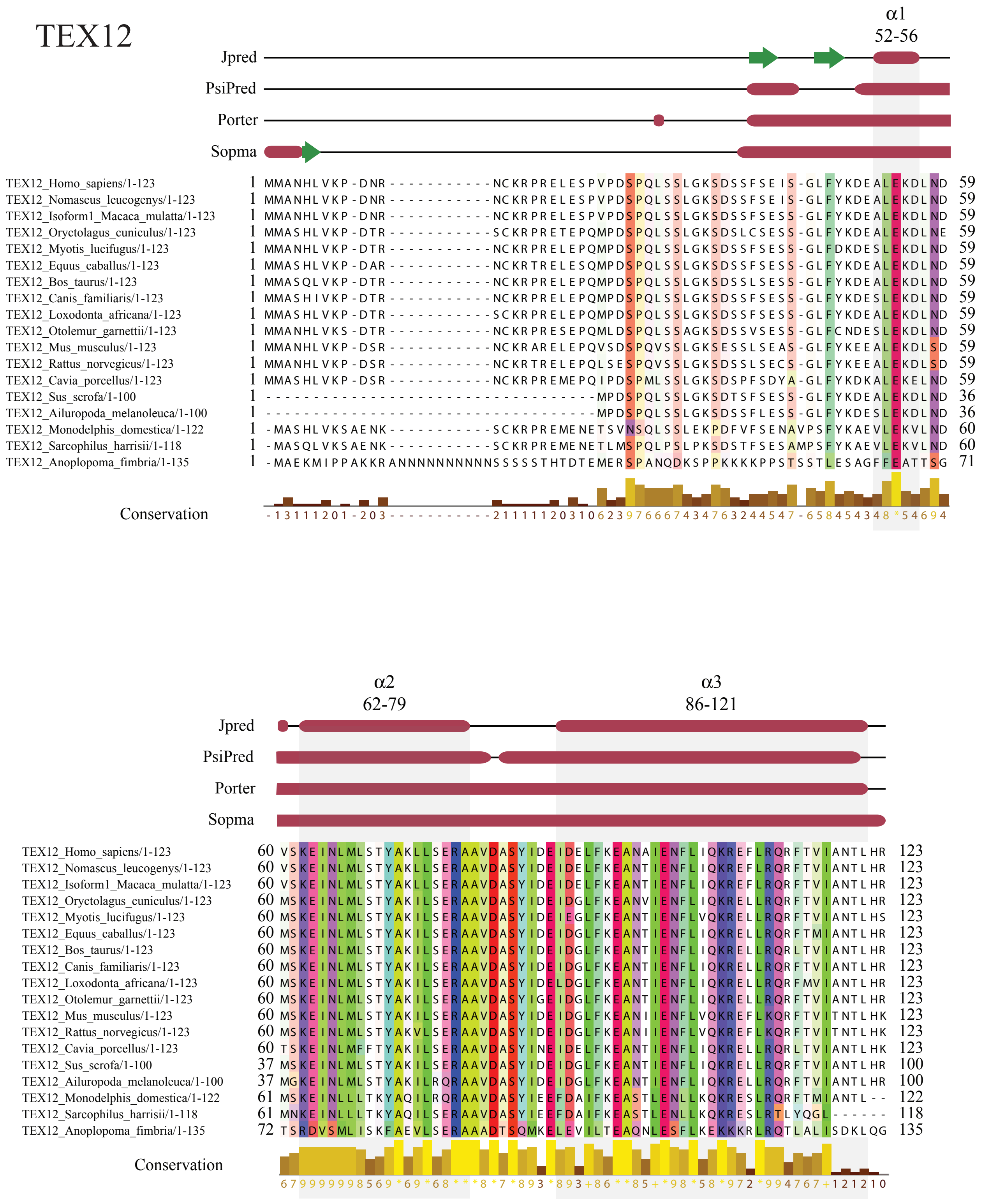


**Figure S2. TEX12 sequence alignment, including secondary structure prediction.** Predicted α-helices and β-sheets are shown for four secondary structure prediction algorithms: Jpred, PsiPred, Porter and Sopma. Annotated helices α1, α2 and α3 relate to the Jpred prediction. Coiled-coil formation is not predicted for TEX12 (JNet: Lupas algorithm).

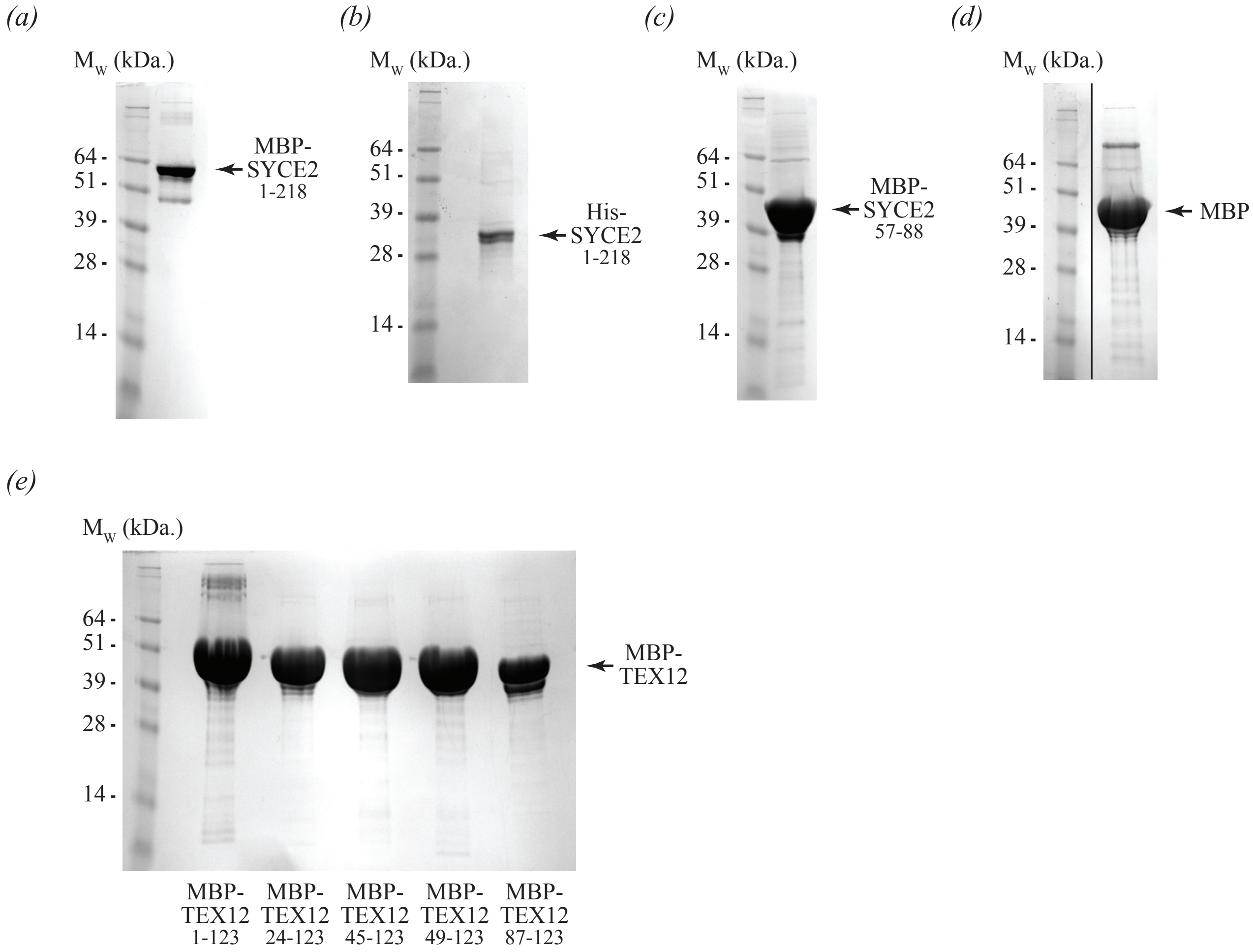


**Figure S3. Purification of SYCE2 and TEX12 constructs for analysis by SEC-MALS.** Coomassie-stained SDS-PAGE of recombinant proteins (a) MBP-SYCE2$_{1\text{-}218}$, (b) His-SYCE2$_{1\text{-}218}$, (c) MBP-SYCE2$_{57\text{-}88}$, (d) free MBP, (e) MBP-TEX12 1-123, 24-123, 45-123, 49-123 and 87-123.

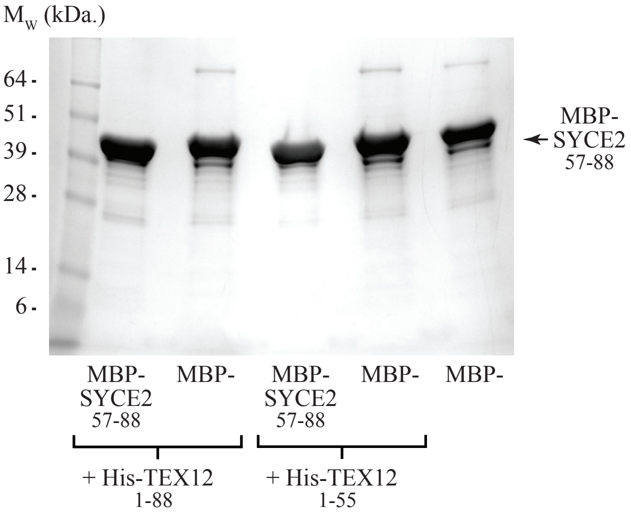


**Figure S4. The N-terminal and central α1-2 regions of TEX12 do not interact with SYCE2.** Amylose pull-down of His-TEX12 1-88 and 1-55 following co-expression with MBP-SYCE2 57-88 and with free MBP, visualised by Coomassie staining. The His-TEX12 1-88 and 1-55 proteins are 14 kDa and 10 kDa respectively.
